# An Investigation into Accumulative Difference Mechanism in Time and Space for Material Removal in Micro-EDM Milling

**DOI:** 10.3390/mi12060711

**Published:** 2021-06-17

**Authors:** Qi Jing, Yongbin Zhang, Lingbao Kong, Min Xu, Fang Ji

**Affiliations:** 1 Shanghai Engineering Research Center of Ultra-Precision Optical Manufacturing, School of Information Science and Technology, Fudan University, Shanghai 200438, China; qjing17@fudan.edu.cn (Q.J.); minx@fudan.edu.cn (M.X.); 2 Institute of Machinery Manufacturing Technology, China Academy of Engineering Physics, Mianyang 621000, China; zcaep6@163.com (Y.Z.); jfang2013@caep.cn (F.J.)

**Keywords:** micro-EDM milling, accumulative difference, material removal, tool electrode wear

## Abstract

In micro-electrical discharge machining (micro-EDM) milling, the cross-section of the microgroove machine is frequently not an ideal rectangle. For instance, there are arc shapes on the bottom and corners, and the sidewall is not steep. The theoretical explanation for this phenomenon is still lacking. In addition to the tip discharge effect, the essential reason is that there is an accumulative difference in time and space during the shape change process of a tool electrode and the microstructure formation on a workpiece. The process parameters are critical influencing factors that determine this accumulative difference. Therefore, the accumulative difference mechanism in time and space is investigated in this paper, and then a theoretical model is developed to simulate the micro-EDM milling process with a straight-line single path. The simulation results for a cylindrical electrode at the two rotational speeds of 0 (nonrotating) and 300 rpm are compared, while the results for a cylindrical electrode and a square electrode at a rotation speed of 0 are also compared to verify that different process parameters generate accumulative differences in the time and space of material removal. Finally, micro-EDM milling experiments are carried out to verify the simulation model. The maximum mean relative deviation between the microgroove profiles of simulation results and those of experiments is 11.09%, and the profile shapes of simulations and experiments have a good consistency. A comparative experiment between a cylindrical electrode and a hollow electrode is also performed, which further verifies the mechanism revealed in the study. Furthermore, the cross-section profile of a microgroove can be effectively controlled by adjusting the process parameters when utilising these accumulative differences through fabricating a microgroove with a V-shaped cross-section by a square electrode and a microgroove with a semi-circular cross-section by a cylindrical electrode. This research provides theoretical guidance for solving the problems of the machining accuracy of detail features in micro-EDM milling, for instance, to machine a microgroove with an ideal rectangular cross-section.

## 1. Introduction

Micro-electrical discharge machining (micro-EDM) is a kind of machining technology that removes materials by means of the extremely high temperature generated by spark discharge. The tool electrode and the workpiece retain a certain gap between them and do not come into contact, and there is no macro-cutting force. Microtool electrodes with a scale of several microns can be fabricated; therefore, the size of machined structures can also reach the level of several microns [1]. This process is particularly suitable for machining both hard-to-cut [2,3,4,5,6] and easily deformed conductive materials [7].

As an electrical machining method, the machining localisation and precision of micro-EDM are very high. There is no stray corrosion, which often appears in micro-electrochemical machining (micro-ECM) [8], when the dielectric fluid material is kerosene or air. Furthermore, the corrosion of machine tools and environmental pollution can also be avoided. There is no burr, which is commonly found in micro-milling [9], at the edge of the machining structures. It is hence more suitable for the processing of microstructures on hard metal materials than micro-milling. By controlling the tool electrode with complex three-dimensional movements, complicated micro three-dimensional structures can be fabricated expediently by micro-EDM milling [10], which is generally difficult to achieve in MEMS, LIGA and other semiconductor processes [11,12]. Laser micromachining technology can also be used for machining conductive materials such as metals. Although they have a similar principle, which is to melt materials and vaporize them through a thermal effect, the verticality of the sidewalls and surface quality of the structures machined by micro-EDM are better than those machined by laser micromachining [13]. In addition, micro-EDM offers a better economy, flexibility and convenience than laser micromachining [14]. Some studies have shown that micro-EDM can also be used to machine other materials such as glass, ceramics and semiconductors by adding an auxiliary electrode or changing the working fluid [15,16]. Furthermore, there are many studies focusing on hybrid micro-EDM, and many significant results have been achieved [17]. Therefore, micro-EDM is playing an increasingly important role in the field of micromachining and has many potential applications.

It is typically difficult to fabricate a forming tool electrode for complex micro three-dimensional structures, and the dimensional accuracy of the forming tool is hard to maintain because of the high tool wear. Hence, die-sinking EDM technology is generally not employed to machine complex structures. Wire electrical discharge grinding (WEDG) was developed to address in situ preparation of microtool electrodes in the 1980s, which improved the machining accuracy and promoted the use of micro-EDM [18]. Subsequently, three-dimensional structures, such as microcavities, were fabricated by controlling the microtool electrode for layered milling [10]. However, problems remain in micro-EDM milling, such as tool-electrode wear. Furthermore, the material removal volume of a workpiece is often very large during micro-EDM milling; thus, the axial wear of the tool electrode is considerable due to the relatively small radial dimension, which not only affects the machining efficiency but also seriously influences the machining accuracy. To solve this problem, some studies have focused on the reduction of electrode wear, and others have focused on compensation strategies [19]. Yu et al. [20] proposed the uniform wear method (UWM) for the machining of micro moulds. This method maintains the original electrode shape and converts the three-dimensional electrode wear to a linear process, which greatly simplifies the compensation strategy. Subsequent research put forward various strategies to advance this method [21]. Yu et al. [22] proposed a combined linear uniform (CLU) method that combined the linear compensation method (LCM) and the UWM. The material removal rate, electrode wear ratio and surface roughness were improved using the proposed method compared to those by the uniform wear method while machining a square cavity with slanted surfaces. Li et al. [23] proposed a compensation method based on the scanned area (BSA) in each machining layer. Three-dimensional microcavities were generated using this method. The machining efficiency was improved, and the tool wear ratio was reduced compared with the UWM and CLU methods. Pei et al. [24] introduced a fixed-length compensation (FLC) method where compensation was applied after a fixed machining length. The dimensional precision and shape accuracy of the machined groove were enhanced by this method.

The above studies are all based on the UWM and assume that the wear of the electrode end is uniform through layered thickness control and path planning. Thus, the axial length wear of the electrode and its compensation are the main focus, and whether there is nonuniform wear of the electrode is usually not considered. In addition, the machining efficiency is limited due to the relatively small monolayer thickness. Therefore, Zhang et al. [25] introduced a large monolayer thickness milling method by modifying the conventional FLC model and achieved higher machining efficiency, precision and quality compared with the layer-by-layer machining method. Zhang et al. [26] developed a two-dimensional geometrical simulation model to predict the machined surface of the tool and the workpiece. They provided a better realisation of a cone-shaped tool and indicated that the cone angle of the electrode was formed through the relative motion of the electrode along the workpiece. Pei et al. [27] proposed an improved fixed-length compensation method that was validated experimentally by replacing a cylindrical tool with a tubular-shaped tool, and a truncated conic tool end was observed in experiments. They also developed an analytical model to relate the truncated conic shape to fixed-length compensation parameters. The model was no longer confined to face milling in these studies, but monolayer thickness was increased so that the electrode sidewall is involved in peripheral milling at the same time, which obviously causes radial wear of the electrode and changes the end of the electrode to a conical or truncated conical shape.

However, other studies have found that the shape of the electrode end is also changed during micro-EDM milling by the nonuniform wear of the electrode. This nonuniform wear will lead to an arc shape of the machined groove profile and significantly affect the machining accuracy during the milling of the cavity. Yan et al. [28] presented an electrode wear compensation method based on a machine vision system. Tool electrode wear can be evaluated directly by this vision system. They found that an arc shape at the end of the tool electrode appeared after just one layer of machining. Pham et al. [29] carried out a number of experiments to analyse the shape change of the electrode. They found that the electrode tended to change during machining towards a constant shape and that the milled groove was an arc shape. Karthikeyan et al. [30] found that the shape of the electrode end changed after channel machining during a study of micro-EDM milling. They thought that the tool rotation motion was a key factor. Nguyen et al. [31] presented geometric models to identify and analyse the error components of the 3D micro-EDM milling process. They found that aside from the inherent machining gap and the unavoidable electrode wear, the corner radius of the virtual electrode in the model was also of prime importance in determining the machining accuracy, which indicated that the rounded shape of the tool edge leads to significant geometrical inaccuracies. However, these studies mainly focused on the influence of various process parameters on the machining results under certain specific situations. The mechanism was rarely discussed and analysed. What is more, a generic solution, which may realise the prediction of micro-EDM milling, has not been proposed yet.

At present, in addition to the requirements of dimensional accuracy for many micro three-dimensional structures, important detailed features in the structures are also needed to meet high accuracy requirements. For instance, a folded waveguide with a rectangular cross-section is a common slow-wave structure of the Terahertz travelling wave tube [32], which has strict accuracy requirements on the cross-section, such as the steepness of the sidewall, the flatness of the bottom and the corner radius of the sidewall and bottom. However, it can be seen from previous studies that the nonuniform wear of the tool electrode made the accuracy of the detailed features worse; for instance, an uneven microcavity bottom and a larger sidewall corner radius and cavity bottom. Although a flatter cavity bottom can be achieved by using a smaller microtool electrode through overlapping moving paths, the size of the corner radius is still hard to control. Moreover, it is also difficult to realise the overlap of the moving electrode paths for some narrow grooves with a large aspect ratio, which is usually machined only by the single-path layered milling method. In this case, accuracy control problems will occur easily if the process parameters are inappropriate. Therefore, the effective control of the nonuniform wear of the tool electrode is of great significance in improving the dimensional accuracy of the micro-detailed features, reduce the overlapping rate of the machining paths during the machining process and thereby improve the machining efficiency.

On the other hand, the cross-sectional profile is not necessarily rectangular for some more complex three-dimensional microstructures. Some cross-sections are semi-circular, such as the electron beam tunnels in Terahertz travelling wave tubes [32]. Some are trapezoidal or even V-shaped profiles, such as surface micro-textures [33] and microflow channels [34]. From this perspective, the nonuniform wear characteristics of the electrode in micro-EDM milling can be used to obtain various groove profiles.

Axinte et al. [35] demonstrated that, despite differences in their nature, many energy beam (EB) controlled-depth machining processes could be modelled using the same mathematical framework in the study of freeform surfaces machining. They have developed approaches and algorithms for the inverse problem to generate freeform surfaces using different EB machining processes and various workpiece target materials [36]. Wan et al. [37,38] proposed a time-variant and space-variant tool influence function (TIF) model in the study of precision optical polishing. In order to work out the dwell time, they used a linear transformation to replace the convolution operation. These studies in other fields make us realise that a generic solution can be explored from this perspective to solve the problem of machining accuracy of microstructures caused by the wear of tool electrode.

Micro-EDM is a “profile-copying” machining method, so the shape of the tool electrode and the shape of the machining structure influence and replicate each other. For example, in the process of microgroove milling with a cylindrical electrode, if the cross-section of the microgroove is not an ideal rectangle, it means that the axial cross-section of the electrode is also no longer an ideal rectangle, which means that there is nonuniform wear at the end of the electrode. A literature review indicates that the mechanism and the influence rules of the factors are still unclear for the nonuniform wear phenomenon of tool electrodes and the corresponding profile imprinted onto the workpiece. Hence, further studies should be undertaken to explain this phenomenon and provide theoretical guidance for solving the problems of the machining accuracy of detail features.

To solve this practical problem, the accumulative difference mechanism in time and space of material removal in micro-EDM milling is analysed and investigated combined with the key process parameters. Then, a micro-EDM simulation model is established to verify the mechanism, and machining experiments are also carried out to demonstrate the validities of the simulation model and the revealed mechanism. In addition, grooves with different cross-sections are generated by experiments through the utilisation of this accumulative difference in time and space. This work provides theoretical guidance for solving the problems of the machining accuracy of detail features in micro-EDM milling, for instance, to machine a microgroove with an ideal rectangular cross-section.

## 2. Accumulative Difference Mechanism in Time and Space for Material Removal

**Figure 1:**
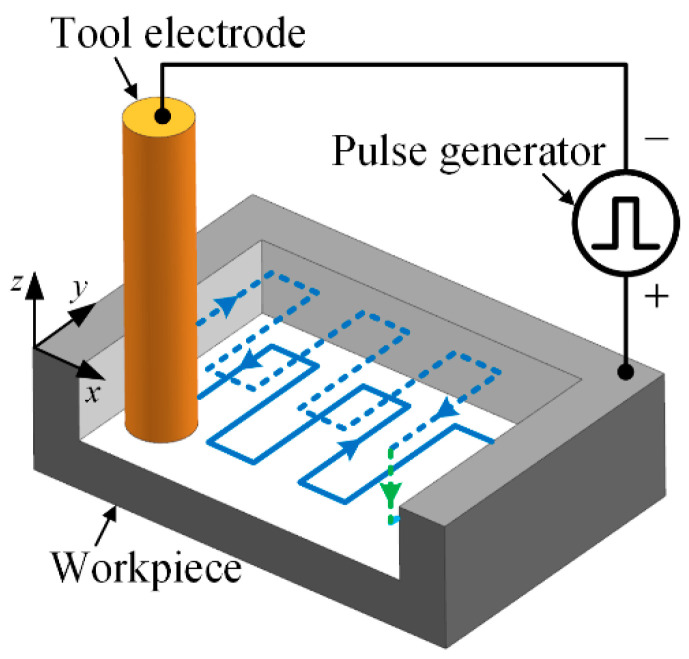
Schematic diagram of micro-EDM milling.

**Figure 2:**
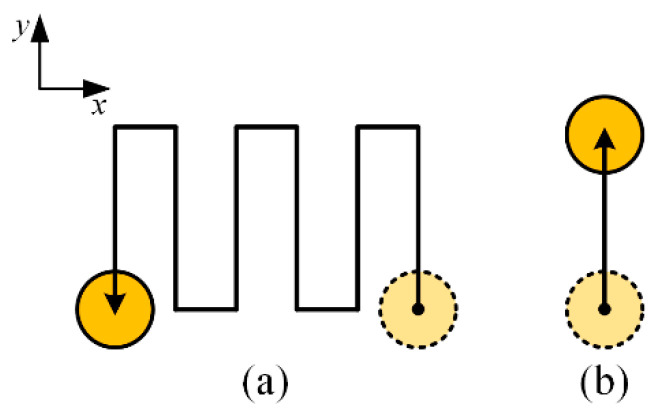
Schematic diagram of (**a**) milling paths in one layer and (**b**) straight-line milling path.

Micro-EDM milling is a kind of EDM method that controls the microtool electrode to move along a certain path, as shown in Figure 1. To keep the radial shape of the electrode unchanged as much as possible, the layered machining method is generally employed. In layered machining, the thickness of one layer is set to be less than the critical discharge gap so that the discharge sparks mainly occur on the bottom end face of the electrode and the opposite face on the workpiece. The sidewall of the electrode hence can be prevented from discharging as much as possible [20]. For each layer of micro-EDM milling, the machining path is shown in Figure 2a. It can be regarded as consisting of many simple paths. The most common single path, named the straight-line path, is shown in Figure 2b. The straight-line path is the basis of micro-EDM milling, which is, therefore, the main study objective in this paper.

**Figure 3:**
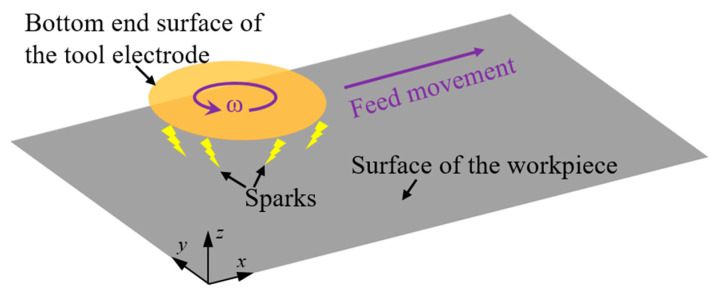
Schematic diagram of the simplified discharge model of micro-EDM milling.

Generally, micro-EDM milling can be simplified as a discharge model between the bottom end surface of the cylindrical electrode and the corresponding surface of the workpiece, as shown in Figure 3. The relative movement between the two surfaces consists of the rotational motion of the cylindrical tool electrode itself and its movement along the machining path. The two surfaces are eroded continuously by the discharging that occurs when a high-frequency pulse breaks down the dielectric fluid.

The coordinate of an arbitrary point on the bottom surface of the tool electrode is set as $\left(x_{T},y_{T}\right)$, and the coordinate of an arbitrary point on the surface of the workpiece is as $\left(x_{W},y_{W}\right)$. After *n* pulses, the *z*-coordinate can be employed to represent the change in the depth direction of an arbitrary point on the tool electrode or the workpiece, as expressed in Equation (1).

(1) $$\{\begin{array}{c} z_{T}\left(x_{T},y_{T}\right)=z_{T0}+\sum_{j=1}^{n}d_{j}\\ z_{W}\left(x_{W},y_{W}\right)=z_{W0}-\sum_{j=1}^{n}h_{j} \end{array}$$

where $z_{T}\left(x_{T},y_{T}\right)$ represents the *z*-direction position of point $\left(x_{T},y_{T}\right)$ and $z_{W}\left(x_{W},y_{W}\right)$ represents the *z*-direction position of point $\left(x_{W},y_{W}\right)$. $z_{T0}$ and $z_{W0}$ are the initial z-direction positions of the tool electrode bottom surface and the workpiece surface, respectively. $d_{j}$ and $h_{j}$ represent the removal depth at this point of the *j*-th pulse on the electrode and the workpiece, respectively. Both $d_{j}$ and $h_{j}$ can be equal to zero.

The tool electrode and the workpiece both undergo electrical discharge erosion as the machining continues, and the materials are removed from both. Thus, the surface morphologies of the tool electrode and the workpiece, which can be represented by $z_{T}$ and $z_{W}$, respectively, are changing continuously. Furthermore, the positional relationship of the spatial point pair that is formed by one point on the electrode and one point on the workpiece also changes with the relative movement between the electrode and workpiece. The spatial position of the pair of erosional craters generated by the effective discharge pulse will hence change over time. Therefore, the formation of the tool electrode and the workpiece profiles is a process in which the erosions of the discharge pulses at different times and spaces accumulate continuously.

During this process, taking the tool electrode as an example, *m* points can be selected randomly on the bottom end surface of the electrode, and a matrix ***D*** can be presented to show their removal depths in time and space after *n* pulses.

$$D(m\times n)=\left[\begin{array}{ccccc} d_{11} & d_{12} & d_{13} & \cdots & d_{1n}\\ d_{21} & d_{22} & d_{23} & \cdots & d_{2n}\\ d_{31} & d_{32} & d_{33} & \cdots & d_{3n}\\ \vdots & \vdots & \vdots & \ddots & \vdots \\ d_{m1} & d_{m2} & d_{m3} & \cdots & d_{mn} \end{array}\right]$$

where the *i*-th row vector $d_{i}=\left[\begin{array}{ccccc} d_{i1}, & d_{i2}, & d_{i3}, & \cdots , & d_{in} \end{array}\right]$ of matrix ***D*** represents the removal depth sequence of point *i*. The *j*-th column vector $d_{j}={\left[\begin{array}{ccccc} d_{1j}, & d_{2j}, & d_{3j}, & \cdots , & d_{mj} \end{array}\right]}^{T}$ of matrix ***D*** represents the removal depth sequence of all the points generated by pulse *j*.

There are many process parameters in micro-EDM, such as parameters that determine the discharge energy per single pulse, including the open-circuit voltage, peak current and pulse width, and other parameters, such as the electrode material, workpiece material, dielectric fluid, machining polarity, rotation speed, geometric shape of the electrode, feed speed, path, pulse frequency, layer thickness and control threshold of the gap voltage. The various combinations of these parameters will result in different material removal volumes per discharge and lead to different spatial positions per discharge where the removal is located, which means that different process parameters determine different matrixes ***D***.

For a certain set of process parameters, the formation of the removal depth sequence ***d****_j_* generated by pulse *j* will be initially influenced in the time domain by the accumulative result of the erosions generated by the previous *j*-1 pulses. Meanwhile, the surface morphologies of the tool electrode and the workpiece change due to the erosion of the *j*-th pulse. The spatial relative position of the tool electrode and workpiece is also changed by the relative movement between them during this time, which will also influence the composition of the next column vector, which is ***d****_j_*_+1_. Because of this accumulative effect in time and space, the accumulative removal amount may be different at different positions of the end surface of the tool electrode, which means that each row of matrix ***D*** has differences. If proper parameters are adopted so that the sum of ***d****_i_* that can be represented by $\sum_{j=1}^{n}d_{ij}$ is approximately equal regardless of how much *i* is, then uniform wear of the electrode can be achieved. The material of the corresponding machining area of the workpiece can also be uniformly removed according to a “profile-copying” micro-EDM characteristic, such as the groove with a rectangular cross-section. However, if the parameters adopted make a relatively large difference in $\sum_{j=1}^{n}d_{ij}$ when *i* is different, then nonuniform wear of the electrode occurs. Likewise, this kind of nonuniform shape change will also be “copied” to the workpiece, resulting in nonuniform material removal of the workpiece, such as a groove with an arc cross-section.

Consequently, this accumulative difference in time and space can be adjusted by adjusting the process parameters. As a result, the wear amount at different positions on the bottom of the electrode and the final machining profile of the workpiece can be effectively controlled.

In the straight-line single path of micro-EDM milling, taking the common cylindrical tool electrode as an example, the different combinations of the feed speed and rotation speed of the electrode can reveal the difference in the spatial point pair relationship between the tool electrode and workpiece. The probability of pulse erosion at the different positions on the bottom surface of the electrode will thus be different. The motion trajectory of different positions on the bottom surface of the cylindrical electrode when the feed direction is in the *x*-direction is expressed by Equation (2).

(2) $$\{\begin{array}{l} x=vt-r\sin\left(\frac{\pi n}{30}t\right)\\ y=r\cos\left(\frac{\pi n}{30}t\right) \end{array}$$

where *x* and *y* are the *x*-coordinate and *y*-coordinate of the point, respectively, *v* is feed speed (μm/s), *t* is the time (s), *r* is the distance between the centre point of the bottom surface of the electrode and the point (μm) and *n* is the rotation speed (rpm).

According to the relationship between the linear feed speed and rotation speed, the motion trajectory of the point that is on the circle with a radius of $r=\frac{30v}{\pi n}$ is cycloid, and the motion trajectory of the other points (except the centre point of the bottom surface of the electrode) is trochoid.

**Figure 4:**
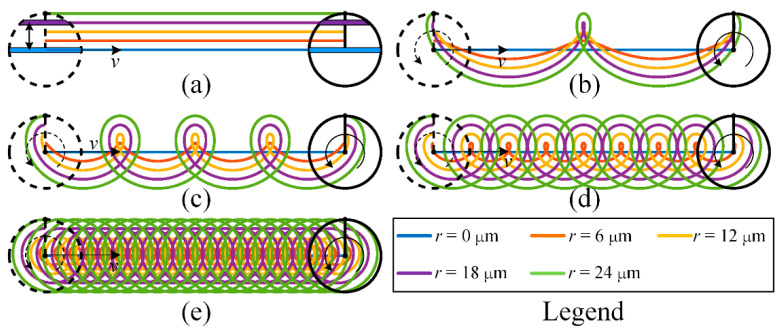
Differences in spatial motion trajectories of different points on the bottom surface of a cylindrical electrode when the rotation speed of the electrode is (**a**) 0; (**b**) 30; (**c**) 60; (**d**) 120; and (**e**) 300 rpm.

The motion trajectories are shown in Figure 4. Figure 4a shows the motion trajectories of the points in different radii on the bottom surface of the electrode when the rotation speed of the electrode is 0, and Figure 4b–e show those when the rotation speed of the electrode is 30, 60, 120 and 300 rpm, respectively. It can be seen from Figure 4a that when the electrode is not rotating, the trajectories are parallel. The chord length of the bottom surface of the electrode along the machining direction varies with its distance from the machining centre axis. Obviously, the smaller the distance between the chord and the machining centre axis is, the longer the chord length is, and the more material removal of the workpiece is. When the electrode rotates, the motion trajectories of the points in different radii are quite different, so the probability of discharge at each point may also be different. When the feed speed remains unchanged, these trajectories become denser with increasing rotation speed, and the difference in the probability of discharge at each point will also change.

Through the above analysis, it can be concluded that the combination of various process parameters in micro-EDM milling will influence the composition of matrix ***D***, which generates the difference in material removal under different parameters. On the other hand, the matrix ***D*** may be different in time and space dimensions for a certain set of process parameters. This is the accumulative difference mechanism in time and space for material removal in micro-EDM milling.

## 3. Modelling and Simulation of Material Removal in Micro-EDM Milling

Micro-EDM is a complicated physical process that is affected by various influencing factors. It is difficult and laborious to simulate the entire micro-EDM milling machining process from the perspective of physical processes [39]. In some previous studies, researchers have proposed geometric simulation models to simulate various EDM processes [40,41,42,43].

**Figure 5:**
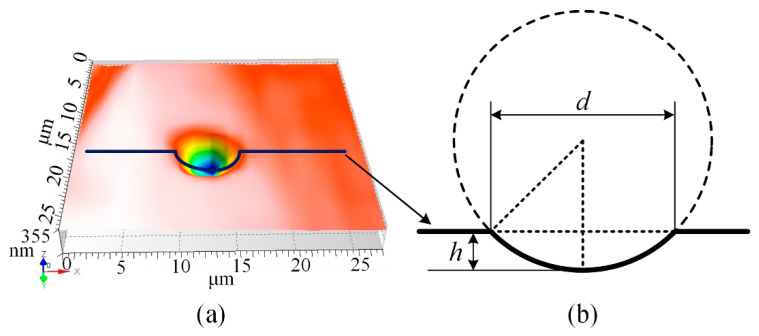
Crater eroded by single discharge: (**a**) actual crater on the workpiece, and (**b**) simplification of crater shape with a spherical crown model.

The profiles of the electrode and workpiece generated in micro-EDM are accumulative results that are the accumulation of each discharge crater eroded by a single discharge pulse [39]. Based on this technique, a geometric simulation method will be employed in this paper [42], in which the single discharge crater eroded by a single discharge pulse is considered the elementary unit to calculate the amount of material removal. A schematic diagram of a single discharge crater is shown in Figure 5. The actual crater on the workpiece shown in Figure 5a can be simplified with a spherical crown model shown in Figure 5b, where *h* is the depth of the crater and *d* is the diameter of the crater.

Therefore, the following assumptions are devised before modelling to simplify the simulation process: (1) the discharge occurs at the points between the tool electrode and the workpiece with the shortest distance that are no more than the critical discharge gap; (2) only one micro-energy electrical pulse is generated at each time step in the simulation; (3) the energy of each effective discharge spark is constant, and the material removal volume per spark is constant; and (4) the influences of the removed debris and the flushing of the dielectric fluid are ignored.

**Figure 6:**
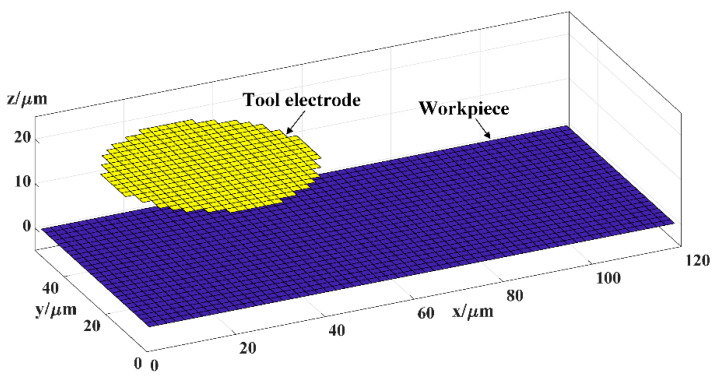
Meshing of the tool electrode and the workpiece.

**Figure 7:**
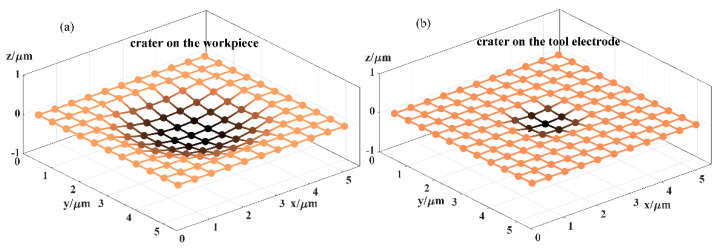
Schematic diagram of a single discharge crater represented by grids: (**a**) crater on the workpiece and (**b**) crater on the tool electrode.

To describe the material removal through calculation, the two geometric models of the tool electrode and the workpiece and the time domain need to be discretised. Hence, the Z-map algorithm [42] is employed to define the geometric models, which represent the bottom surface of the tool electrode and the upper surface of the workpiece, and a set of square grids with the same size are used to mesh them, as shown in Figure 6. Therefore, the workpiece coordinate system is fixed, and the relative motion during machining can be expressed as the movement of the tool electrode coordinate system in the workpiece coordinate system. Therefore, the X-Y coordinates of the Z-map node of the tool electrode in the workpiece coordinate system will change with the movement and rotation of the tool electrode. The shape of a single discharge crater on the tool electrode and the workpiece can also be represented by a Z-map, as shown in Figure 7. In the time domain, the simulation time step Δt is determined by the pulse frequency *f* according to assumption (2), as shown in Equation (3).

(3) $$\Delta t=\frac{1}{f}$$

**Figure 8:**
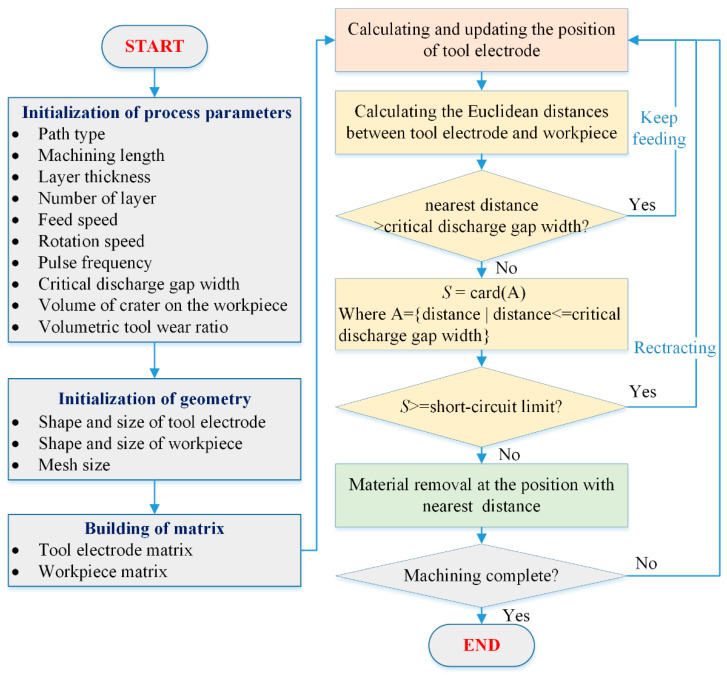
Schematic flowchart of the micro-EDM milling process simulation model.

Thus, the entire calculation and simulation processes of micro-EDM milling are described by the following steps: (1) calculating and updating the position coordinates of the grid node of the tool electrode in the workpiece coordinate system according to the state of motion of the electrode; (2) determining whether discharge occurs and finding the location where the discharge occurs on both the tool electrode and workpiece; and (3) generating the single discharge craters at the corresponding locations for the tool electrode and the workpiece, respectively. The three steps are repeated until the set goals are reached. The flow chart of the entire simulation process is shown in Figure 8.

As shown in the simulation flow chart, there may be three situations for step (2) according to assumption (1):

(a) The distance between the nearest point pair is larger than the critical discharge gap, which means that material removal is not generated by the current pulse during this time step. This is the open-circuit state in micro-EDM.
(b) There are *S* point pairs whose distances are less than or equal to the critical discharge gap, but *S* is larger than or equal to the number of short-circuit limits. Material removal is not generated by the current pulse in this time step either, and the tool electrode will retract along the original path. This is the short-circuit state in micro-EDM.
(c) Conversely, if *S* is less than the number of short-circuit limits, the point pair whose distance is the shortest will be selected as the locations where the discharge occurs. If there are multiple point pairs that have the same shortest distance, a pair will be randomly selected from them. Material removal is generated by the current pulse during this time step. This is the effective-spark state in micro-EDM. The Euclidean distances of the point pairs between the tool electrode and the workpiece can be calculated by Equation (4).

(4) $$G\left(M\times N\right)=\sqrt{\left(\begin{array}{cccc} {\left\|T_{1}\right\|}_{2}{}^{2} & {\left\|T_{1}\right\|}_{2}{}^{2} & \cdots & {\left\|T_{1}\right\|}_{2}{}^{2}\\ {\left\|T_{2}\right\|}_{2}{}^{2} & {\left\|T_{2}\right\|}_{2}{}^{2} & \cdots & {\left\|T_{2}\right\|}_{2}{}^{2}\\ \vdots & \vdots & \ddots & \vdots \\ {\left\|T_{M}\right\|}_{2}{}^{2} & {\left\|T_{M}\right\|}_{2}{}^{2} & \cdots & {\left\|T_{M}\right\|}_{2}{}^{2} \end{array}\right)+\left(\begin{array}{cccc} {\left\|W_{1}\right\|}_{2}{}^{2} & {\left\|W_{2}\right\|}_{2}{}^{2} & \cdots & {\left\|W_{N}\right\|}_{2}{}^{2}\\ {\left\|W_{1}\right\|}_{2}{}^{2} & {\left\|W_{2}\right\|}_{2}{}^{2} & \cdots & {\left\|W_{N}\right\|}_{2}{}^{2}\\ \vdots & \vdots & \ddots & \vdots \\ {\left\|W_{1}\right\|}_{2}{}^{2} & {\left\|W_{2}\right\|}_{2}{}^{2} & \cdots & {\left\|W_{N}\right\|}_{2}{}^{2} \end{array}\right)-2\times TW^{T}}$$

where ***T**_i_* represents the *i*-th row of matrix and T(M×3) are the three-dimensional coordinates of all grid nodes on the tool electrode. $W_{i}$ represents the *i*-th row of matrix W(N×3), and W(N×3) are the three-dimensional coordinates of all grid nodes on the workpiece. G(M×N) is the distance matrix of point pairs between the tool electrode and the workpiece.

**Figure 9:**
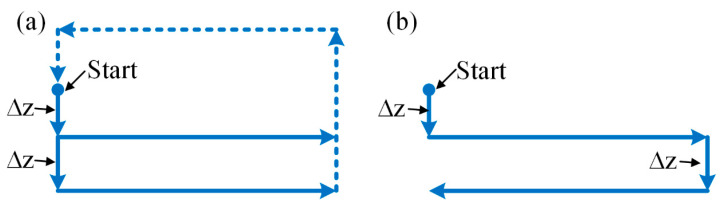
Schematic diagram of two types of paths in straight-line path milling: (**a**) unidirectional path and (**b**) reciprocating path.

Two types of electrodes, cylindrical electrodes and square electrodes, which are common in micro-EDM, are selected in the simulation experiment. There are two machining modes—the rotating and nonrotating mode—for the cylindrical electrode and only the nonrotating mode for the square electrode. In addition, there are also two types of paths—a unidirectional and reciprocating path—in straight-line path milling, as shown in Figure 9. The simulation parameters are presented in Table 1. The critical discharge gap, the shapes of the single discharge craters on the tool electrode and workpiece and the volumetric tool wear ratio depend on the machining parameters, which are determined through preliminary experiments. The electrical parameters and materials of the preliminary experiments are the same as those in Section 4.

**Figure 10:**
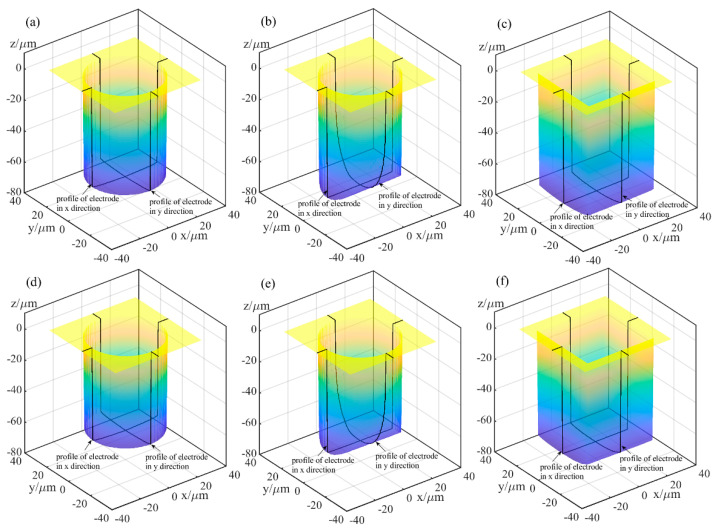
Simulation machining results of the tool electrodes: (**a**) cylindrical electrode-rotating mode-unidirectional path; (**b**) cylindrical electrode-nonrotating mode-unidirectional path; (**c**) square electrode-nonrotating mode-unidirectional path; (**d**) cylindrical electrode-rotating mode-reciprocating path; (**e**) cylindrical electrode-nonrotating mode-reciprocating path; (**f**) square electrode-nonrotating mode-reciprocating path.

**Figure 11:**
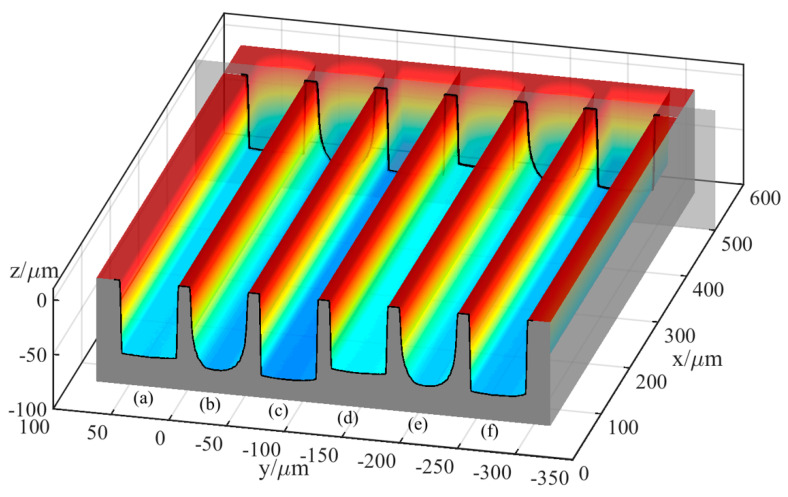
Simulation machining results of the microgrooves: (**a**) cylindrical electrode-rotating mode-unidirectional path; (**b**) cylindrical electrode-nonrotating mode-unidirectional path; (**c**) square electrode-nonrotating mode-unidirectional path; (**d**) cylindrical electrode-rotating mode-reciprocating path; (**e**) cylindrical electrode-nonrotating mode-reciprocating path; (**f**) square electrode-nonrotating mode-reciprocating path.

Figure 10 and Figure 11 show the simulation machining results of the tool electrodes and microgrooves, respectively. It can be seen from Figure 10a,d and Figure 11a,d that the sidewalls of the microgrooves are almost perpendicular to the bottom surface, and the bottom surface is relatively flat. In addition, there is no obvious nonuniform wear on the end of the cylindrical tool electrode. Figure 10b,e and Figure 11b,e are the simulation machining results for the cylindrical tool electrode in nonrotating mode. The results show that the cross-sections of the microgrooves are no longer an approximate rectangle but a typical arc shape. Moreover, there are two clearly different profiles for the cylindrical tool electrode in two directions. In the direction parallel to the machining direction, the profile of the central cross-section is approximately rectangular, while it is also a typical arc shape similar to the cross-section of the microgroove in the direction that is perpendicular to the machining direction. Figure 10c,f and Figure 11c,f show the simulation machining results of the square tool electrode in nonrotating mode. As shown in Figure 10c,f and Figure 11c,f, although the electrodes do not rotate, the sidewalls of the microgrooves are perpendicular to the bottom surface, and the cross-sections are approximately rectangular. There is little difference between the central cross-sections of the square tool electrode in the two directions that are perpendicular and parallel to the machining direction, but there is no noteworthy arc phenomenon.

The simulation results indicate that different rotation speeds and different electrode shapes generate different microgrooves, which demonstrates that the accumulative difference in the time and space of material removal is a critical reason for the nonuniform wear of the electrode and the generation of the corresponding cross-sectional profile of the microgroove. Furthermore, simulation experiments can be carried out based on the established simulation model to predict the machining profile so that the process parameters can be pre-determined, and the regulation and control of this accumulative difference can be achieved.

## 4. Experimental Design and Studies

### 4.1. Experimental Apparatus and Materials

**Figure 12:**
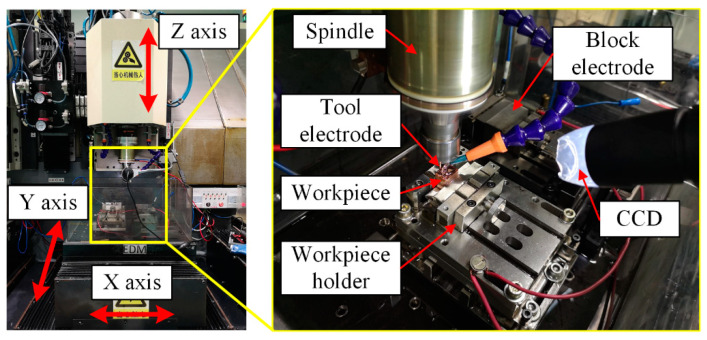
Photo of the experimental apparatus.

The experiments were performed on a micro-EDM machine tool named μEM-200CDS2 (self-developed, Mianyang, China), as shown in Figure 12. Its maximum travelling length is 200 (*X*-axis) × 100 (*Y*-axis) × 200 mm (*Z*-axis). The resolution is 0.1 μm, and the repeatability is 1 μm for all axes. The tool electrode is clamped on a spindle, and the workpiece is fixed on a workpiece holder, which can also level the workpiece. The tool electrode and the workpiece are both copper, and the material properties are listed in Table 2. EDM oil is selected as the dielectric fluid.

### 4.2. Experimental Procedure and Conditions

**Figure 13:**
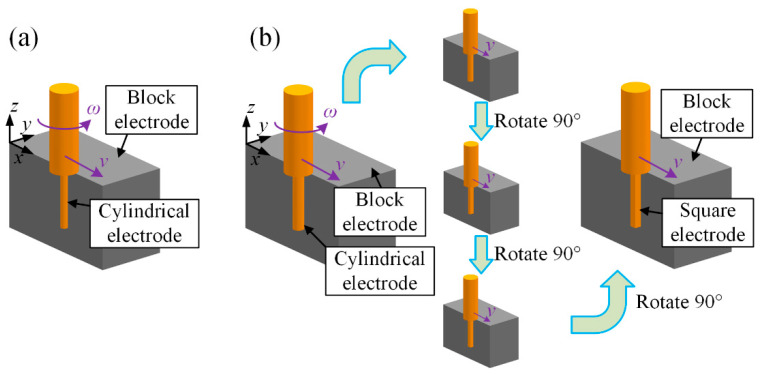
Schematic diagram of the in situ fabrication of the tool electrode: (**a**) cylindrical electrode and (**b**) square electrode.

**Figure 14:**
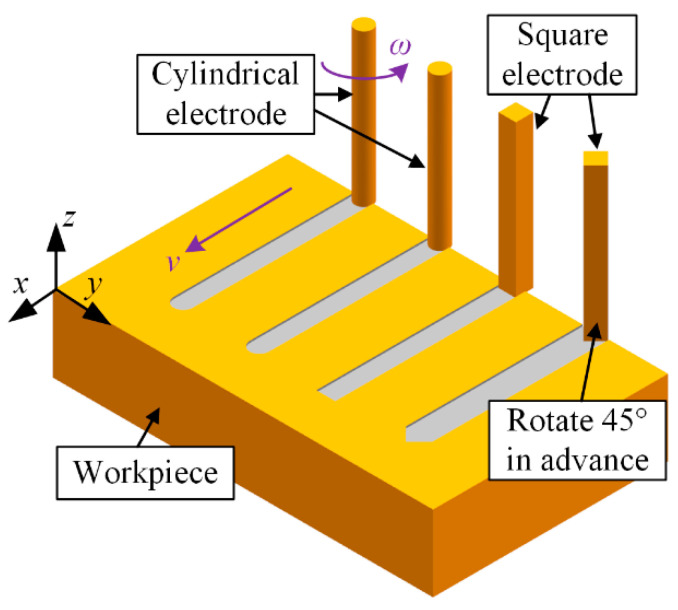
Schematic diagram of the machining process.

First, a set of verification experiments (named Exp. 1) of the mechanism and simulation model was undertaken. A total of six tool electrodes were fabricated in situ using the block electric discharge grinding (block-EDG) method, as shown in Figure 13. Among them, there are four cylindrical electrodes with diameters of 46 μm and two square electrodes with edge lengths of 46 μm. These tool electrodes were employed to perform straight-line path micro-EDM milling on the workpiece in sequence. The schematic diagram of the process is shown in Figure 14. The detailed process parameters are listed in Table 3, which correspond with the simulation parameters in Section 3.

Two cylindrical electrodes with a diameter of 0.8 mm and two hollow electrodes with a diameter of 0.8 mm were fabricated in situ, and straight-line path micro-EDM milling was also performed (named Exp. 2). The processing parameters are listed in Table 4.

Finally, two application experiments of the material removal mechanism in time and space (named Exp. 3) were undertaken. A cylindrical electrode with a diameter of 46 μm and a square electrode with edge lengths of 46 μm were fabricated in situ again. For the cylindrical electrode, the number of machining layers is decreased. The square electrode was rotated 45 degrees before milling, as shown in Figure 14. The other parameters are the same as those in Exp. 1. The processing parameters are listed in Table 5.

### 4.3. Measurements and Observation

The panoramas of the machined microgrooves were observed using a scanning electron microscope (SEM, Zeiss, Jena, Germany). The tool electrodes and the entrance profiles of the microgrooves were observed using a digital microscope (VHX-6000, Keyence, Osaka, Japan). The data of the profile curves can be acquired through image processing, which can be compared with the simulation results.

## 5. Results and Discussion

**Figure 15:**
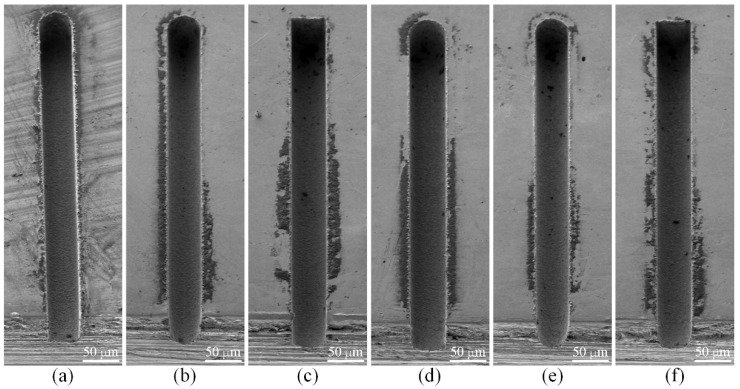
SEM images of the six microgrooves in Exp. 1: (**a**) cylindrical electrode-rotating mode-unidirectional path; (**b**) cylindrical electrode-nonrotating mode-unidirectional path; (**c**) square electrode-nonrotating mode-unidirectional path; (**d**) cylindrical electrode-rotating mode-reciprocating path; (**e**) cylindrical electrode-nonrotating mode-reciprocating path; (**f**) square electrode-nonrotating mode-reciprocating path.

**Figure 16:**
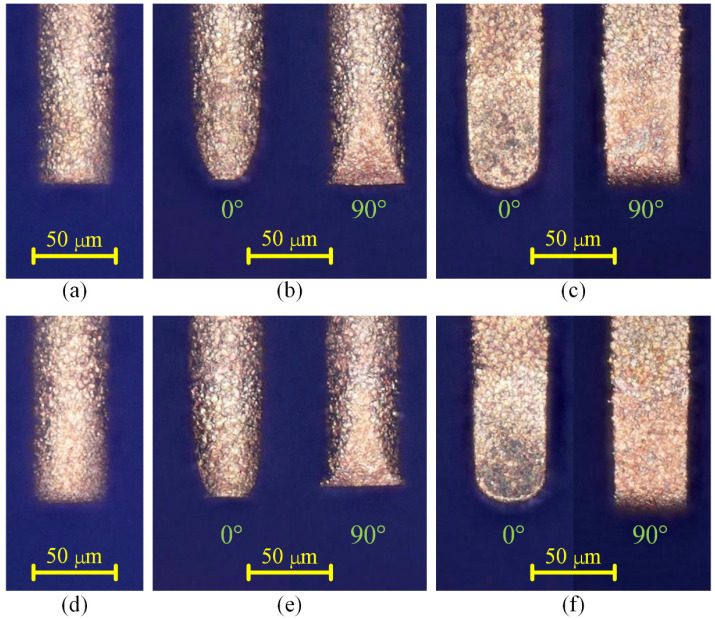
Optical images of the six electrodes in Exp. 1: (**a**) cylindrical electrode-rotating mode-unidirectional path; (**b**) cylindrical electrode-nonrotating mode-unidirectional path; (**c**) square electrode-nonrotating mode-unidirectional path; (**d**) cylindrical electrode-rotating mode-reciprocating path; (**e**) cylindrical electrode-nonrotating mode-reciprocating path; (**f**) square electrode-nonrotating mode-reciprocating path.

**Figure 17:**
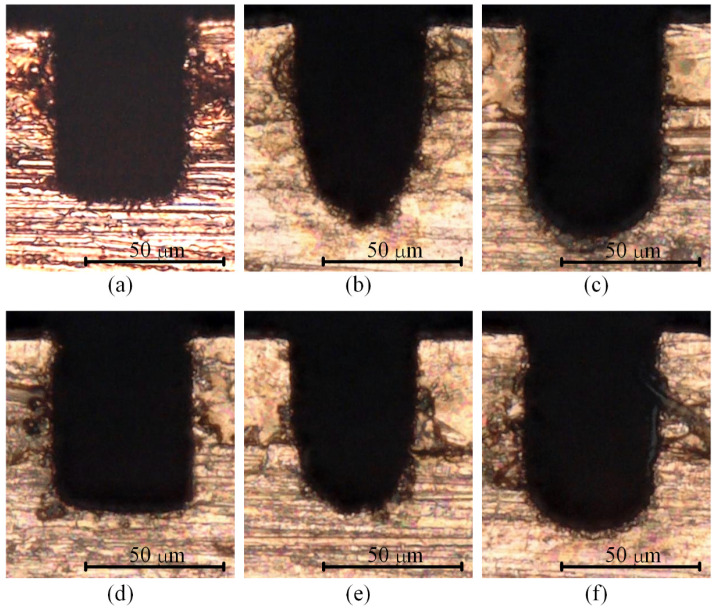
Optical images of the six entrance profiles of the microgrooves in Exp. 1: (**a**) cylindrical electrode-rotating mode-unidirectional path; (**b**) cylindrical electrode-nonrotating mode-unidirectional path; (**c**) square electrode-nonrotating mode-unidirectional path; (**d**) cylindrical electrode-rotating mode-reciprocating path; (**e**) cylindrical electrode-nonrotating mode-reciprocating path; (**f**) square electrode-nonrotating mode-reciprocating path.

Figure 15 shows the SEM images of the six microgrooves in Exp. 1. From the SEM images of the overall morphology, it can be seen that any cross-section of each microgroove in the direction that is perpendicular to the machining direction is highly consistent, which is also very consistent with the simulation results. Figure 16 and Figure 17 show the optical images of the six electrodes and six entrance profiles of the microgrooves in Exp. 1, respectively. In Figure 16, 0 degrees represents that the observation direction of the electrode is parallel to the machining direction, so the observed cross-section of the electrode is perpendicular to the machining direction, and 90 degrees represents that the observation direction is perpendicular to the machining direction, so the observed cross-section of the electrode is parallel to the machining direction, which is also applicable to the following figures in this paper.

It can be seen in Figure 16a,d and Figure 17a,d that when the cylindrical tool electrode rotates during machining, the end face of the cylindrical tool electrode is flat, and the sidewall of the machined microgroove is perpendicular to the bottom surface, which is also relatively flat. Figure 16b,e and Figure 17b,e show that when the cylindrical tool electrode does not rotate during machining, the cross-section of the machined microgroove is a typical arc shape. Furthermore, there are two clearly different profiles of the cylindrical tool electrode in two directions. The profile of the central cross-section is approximately rectangular in the direction that is parallel to the machining direction, while it is also a typical arc shape similar to the cross-section of the microgroove in the direction that is perpendicular to the machining direction. Figure 16c,f and Figure 17c,f show that when the square tool electrode does not rotate during machining, the sidewall of the machined microgroove is perpendicular to the bottom surface, but the bottom surface is not as flat as those in Figure 17a,d, and a slight arc shape appears. In addition, the profile of the central cross-section of the electrode is approximately rectangular in the direction that is parallel to the machining direction, while it is also a slight arc shape that is similar to the cross-section of the microgroove in the direction that is perpendicular to the machining direction.

**Figure 18:**
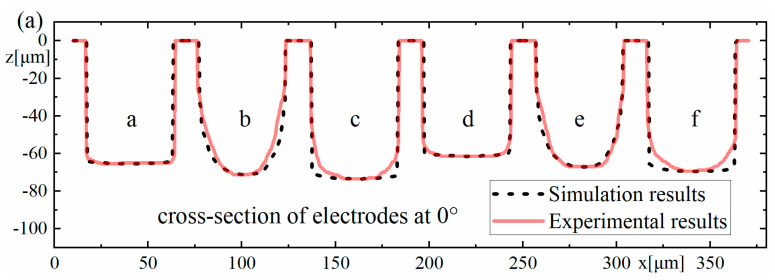

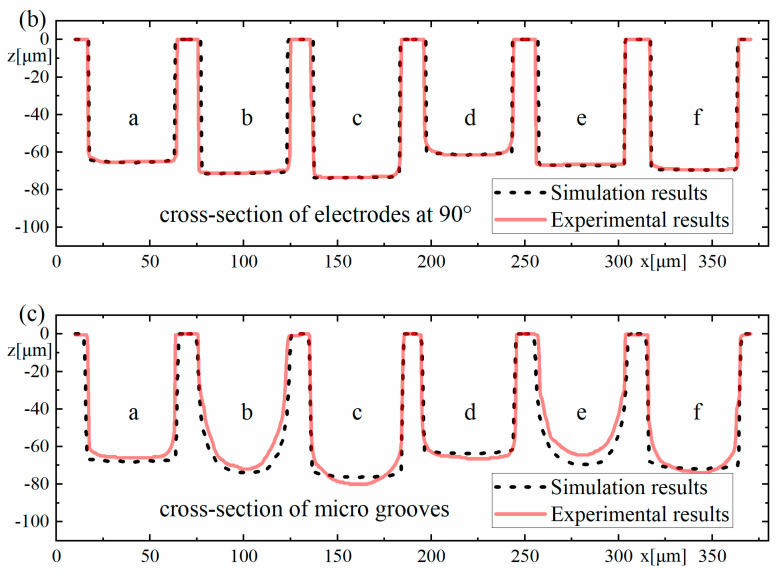
Comparison of the experimental results for Exp. 1 and the simulation results: (**a**) cross-section of electrodes at 0°, (**b**) cross-section of electrodes at 90°, and (**c**) cross-section of microgrooves.

The experimental results and the simulation results are compared together, as shown in Figure 18, which shows that the experimental results agree well with the simulation results overall. The depths of the machined microgrooves in the reciprocating path are slightly less than those in the unidirectional path, which also indicates that there is an accumulative difference in material removal due to the difference in the spatial relative motion relationship. In the machining results for the square electrode, the sidewall of the microgroove is vertical, which is consistent with the simulation. However, there is a certain difference between the bottom surfaces in the experiment and the simulation. Nonetheless, this is only a difference between the bottom surfaces, while the sidewall is still vertical, which is quite different from the machining result when the cylindrical electrode does not rotate during machining. Conversely, when the cylindrical tool electrode does not rotate during machining, as in Exp. 1 b and Exp. 1 e, as shown in Figure 18, the experimental profiles and the simulation profiles are approximately consistent. It should be noted that the debris removal condition will be not ideal if the process parameters are improper in the actual machining process [44], while the simulation is based on the assumption that the debris removal condition has no effect on the machining process.

In the experimental process, the machining process is very stable, and there are almost no abnormal discharges and short circuits when the cylindrical electrode rotates at high speed or does not rotate. This shows that the debris removal condition under these two conditions has no significant impact on the machining process, which satisfies the assumption in the simulation. It is easy to understand that a higher rotation speed can quickly take away the discharge debris. For the cylindrical electrode in nonrotating mode, the tool electrode and microgroove become arc-shaped quickly due to the accumulative difference in time and space, as shown in Figure 16b,e and Figure 17b,e, which makes the working fluid take away the debris more unimpeded. However, for the square electrode in nonrotating mode, the tool electrode shape should remain unchanged from the perspective of the accumulative difference in time and space, as shown in Figure 10c,f. Actually, there is almost no channel for debris to leave the machining gap because the square electrode does not rotate, which is bound to cause a lot of abnormal discharge and short circuit states. This is the reason why the bottom surface in machining results is arc-shaped, which is different from the simulation results. Furthermore, it is why a higher rotation speed and the nonrotating mode are chosen for the verification of the simulation model.

By subtracting the *z*-coordinate value *z*_e_ of the experimental profile from the *z*-coordinate value *z*_s_ of the simulated profile in Figure 18c, the maximum deviation value, the average deviation value of each group and the percentage of average deviation relative to the maximum depth of the corresponding microgroove can be obtained, as shown in Table 6.

**Figure 19:**
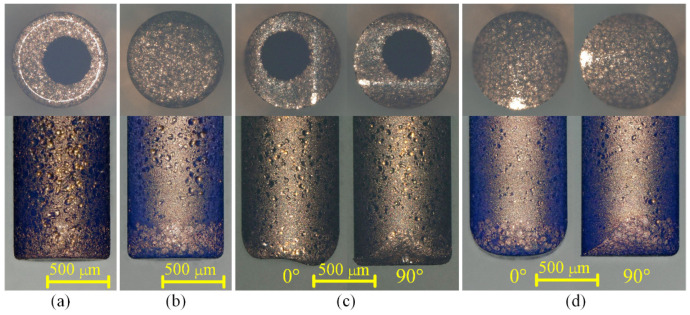
Optical images of the four electrodes in Exp. 2: (**a**) hollow electrode-rotating mode; (**b**) cylindrical electrode-rotating mode; (**c**) hollow electrode-nonrotating mode; (**d**) cylindrical electrode-nonrotating mode.

**Figure 20:**
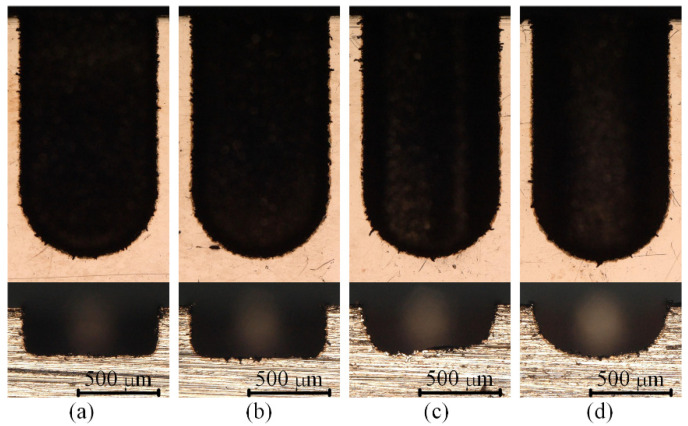
Optical images of the top view and back view of the four grooves in Exp. 2: (**a**) hollow electrode-rotating mode; (**b**) cylindrical electrode-rotating mode; (**c**) hollow electrode-nonrotating mode; (**d**) cylindrical electrode-nonrotating mode.

Figure 19 and Figure 20 show the optical images of the four electrodes and the top view and back view of the four grooves in Exp. 2, respectively. It can be seen from Figure 19a,b and Figure 20a,b that whether using a cylindrical electrode or a hollow electrode, when the electrode rotates during machining, the sidewall of the machined groove is vertical to the bottom surface, and the bottom surface is very flat. The end face of the tool electrode is also flat. Figure 19c and Figure 20c show that when the hollow tool electrode does not rotate during machining, the cross-section of the machined groove is a unique arc shape, which is asymmetric. The profile of the central cross-section is also a unique arc shape that is similar to the cross-section of the groove in the direction that is perpendicular to the machining direction, which is asymmetric as well. It is worth noting that when observing the end surface of the hollow electrode, it can be seen that the hole is eccentric in the direction that is perpendicular to the machining direction. Hence, the machining depth is deeper on the side farther from the hole, and the machining depth is shallower on the closer side to the hole. As a result, the cross-section of the groove and central cross-section of the hollow electrode in the direction that is perpendicular to the machining direction are all asymmetric. Figure 19d and Figure 20d show that when the cylindrical tool electrode does not rotate during machining, the cross-section of the machined groove is a typical arc shape, which is symmetric. The profile of the central cross-section is also a typical arc shape that is similar to the cross-section of the groove in the direction that is perpendicular to the machining direction, which is symmetric as well. The difference in the groove profiles between Figure 20c,d is due to the end face of the hollow electrode lacking a part with respect to the cylindrical electrode. However, the machined groove profiles are very similar to each other as well when both of the electrodes rotate at a certain speed during micro-EDM milling, as shown in Figure 20a,b, which further demonstrates that different electrode shapes can generate accumulative differences in material removal, and this difference can be controlled by adjusting process parameters such as the rotation speed.

**Figure 21:**
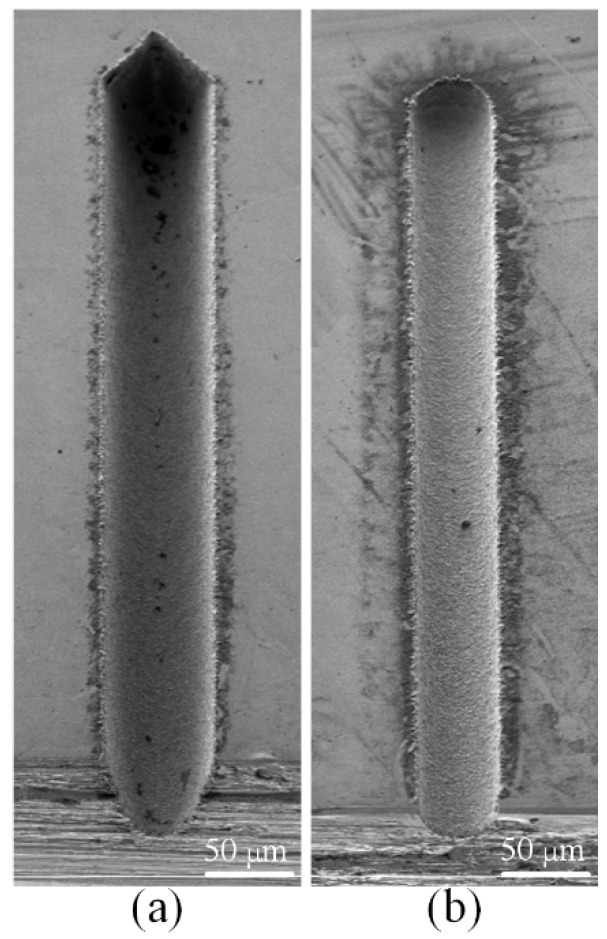
SEM images of the two microgrooves in Exp. 3: (**a**) square electrode-rotate 45° in advance-nonrotating mode; (**b**) cylindrical electrode-nonrotating mode.

**Figure 22:**
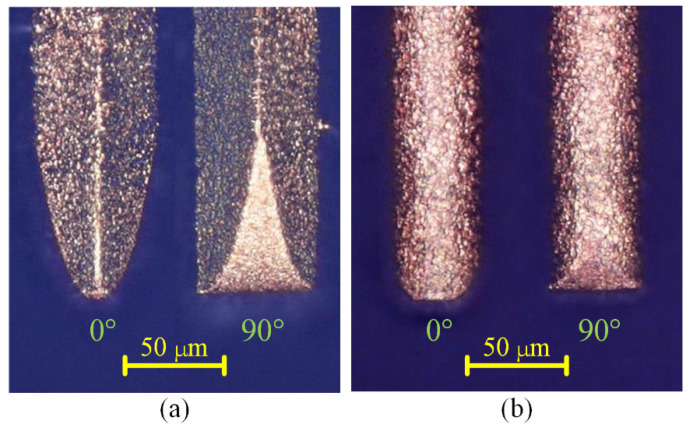
Optical images of the two electrodes in Exp. 3: (**a**) square electrode-rotate 45° in advance-nonrotating mode; (**b**) cylindrical electrode-nonrotating mode.

**Figure 23:**
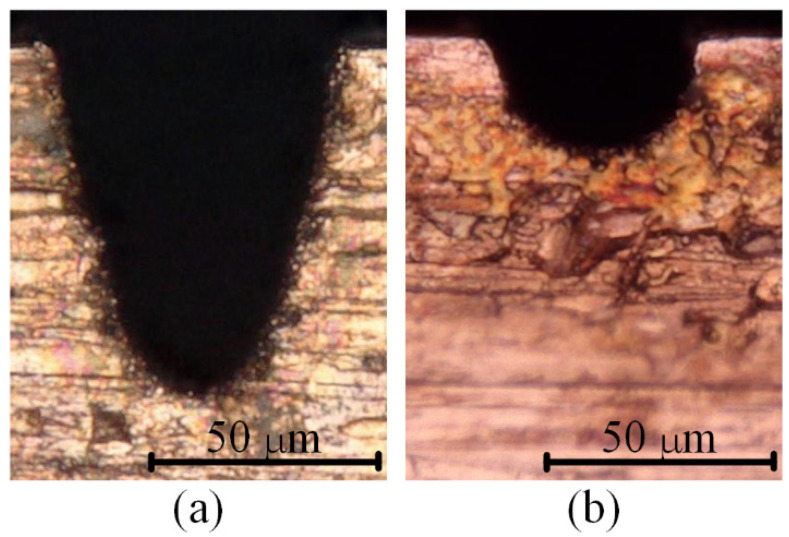
Optical images of the two entrance profiles of the microgrooves in Exp. 3: (**a**) square electrode-rotate 45° in advance-nonrotating mode; (**b**) cylindrical electrode-nonrotating mode.

Figure 21 shows the SEM images of the two microgrooves in Exp. 3. It can also be seen from the SEM images of the overall morphology that any cross-section of each microgroove in the direction that is perpendicular to the machining direction is highly consistent. The length-width ratio of the microgroove is up to 10:1, which also indicates that a microgroove with a special-shaped cross-section and a large length-width ratio can be machined by a simple common electrode through utilising this mechanism so as to avoid the machining error caused by the wear of the forming tool electrode. Figure 22 and Figure 23 show the optical images of the two electrodes and two entrance profiles of the microgrooves in Exp. 3, respectively.

It can be seen in Figure 22a and Figure 23a that when the square electrode was rotated 45 degrees in advance and then micro-EDM milling was performed in nonrotating mode on the workpiece, the cross-section of the microgroove was similar to a V-shape. The profile of the central cross-section is also a V-shape that is similar to the cross-section of the microgroove in the direction that is perpendicular to the machining direction, and it is approximately rectangular in another direction. It can be seen in Figure 22b and Figure 23b that when the cylindrical tool electrode does not rotate during machining, the cross-section of the machined microgroove is an approximately semi-circular shape because of the decrease in the number of machining layers.

**Figure 24:**
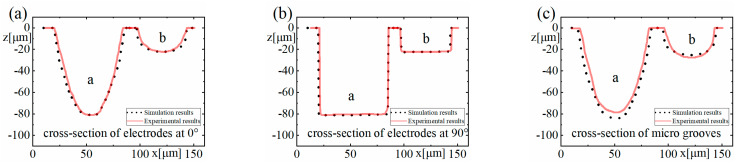
Comparison of the experimental results for Exp. 3 and the simulation results: (**a**) cross-section of electrodes at 0°, (**b**) cross-section of electrodes at 90°, and (**c**) cross-section of microgrooves.

The experimental results are also compared to the simulation results, as shown in Figure 24, which shows that the experimental results agree well with the simulation results. By subtracting the *z*-coordinate value *z*_e_ of the experimental profile from the *z*-coordinate value *z*_s_ of the simulated profile in Figure 24c, the maximum deviation value, the average deviation value of each group and the percentage of average deviation relative to the maximum depth of the corresponding microgroove can be obtained, as shown in Table 7.

## 6. Conclusions

In this work, the nonuniform wear of electrodes and the phenomenon of arc shape appearing on the corresponding workpieces in micro-EDM milling were explained by the accumulative difference mechanism in time and space for material removal. Then, a simulation model was established to simulate straight-line single path micro-EDM milling, and experiments on the straight-line single path of micro-EDM milling were also undertaken. This work provides theoretical guidance for solving the problems of the machining accuracy of detail features, for instance, to machine a microgroove with an ideal rectangular cross-section. The following conclusions can be drawn from the results:

(1) The various combinations of process parameters in micro-EDM milling may lead to accumulative differences in the material removal at the tool electrode and the workpiece. In addition, for a certain set of process parameters, the material removal at the tool electrode and the workpiece may also have accumulative differences in both time and space dimensions, which may cause nonuniform wear of the tool electrode and thereby have an impact on the corresponding profile of the structure on the workpiece.
(2) The established simulation model provides a good verification of the existence of this accumulative difference caused by different process parameters. Furthermore, the maximum mean relative deviation between the microgroove profiles of simulation results and those of experiments is 11.09%, and the profile shapes of simulations and experiments have a good consistency. Therefore, the established simulation model is capable of predicting the machining profile so that the proper process parameters can be pre-determined, and the modification and control of this accumulative difference can be achieved.
(3) The experimental results not only demonstrate the validities of the simulation model and the mechanism revealed but also verify that microgrooves with different cross-sections can be generated through the utilisation of this accumulative difference in time and space.

There are still some problems needing further research. For example, the debris removal condition may be terrible under some process parameters, which may also lead to the nonuniform wear of the tool electrode. Moreover, this will be coupled with the accumulative difference in time and space, which is complicated. Therefore, the influence of process parameters on chip removal will be modelled in the future, which can be introduced into the simulation model in this paper as a factor to provide a more complete model for the explanation of the nonuniform wear of the tool electrode in micro-EDM milling.

In fact, any machining method can be regarded as an accumulative process in time and space, whether it is subtractive manufacturing or additive manufacturing. The accumulative difference mechanism in time and space for material removal in micro-EDM milling proposed in this paper is a generic idea, which can be applied to some similar machining methods, such as pulsed laser machining, water jet machining.

## Figures and Tables

**Table 1:** Simulation parameters.

Parameters	Serial Number					
	a	b	c	d	e	f
Tool type	cylindrical		square	cylindrical		square
Rotation speed (rpm)	300	0		300	0	
Path type	unidirectional path			reciprocating path		
Tool diameter or edge length (μm)				46		
Feed speed (μm/s)				30		
Mesh size (μm)				0.5		
Machining length (μm)				500		
Layer thickness (μm)				1		
Number of layers				150		
Pulse frequency (MHz)				0.67		
Critical discharge gap width (μm)				2		
Volume of crater on the workpiece (μm^3^)				2.4066		
Volumetric tool wear ratio				0.082		

**Table 2:** Material properties.

Physical Properties (20 °C)	Copper
Melting point (**°C**)	1083
Boiling point (**°C**)	2600
Specific heat capacity (J/(kg·K))	400
Thermal conductivity (W/(m·K))	385

**Table 3:** Machining parameters for Exp. 1.

Parameters	Serial Number					
	a	b	c	d	e	f
Tool type	cylindrical		square	cylindrical		square
Rotation speed (rpm)	300	0		300	0	
Path type	unidirectional path			reciprocating path		
Tool diameter or edge length (μm)				46		
Feed speed (μm/s)				30		
Machining length (μm)				500		
Layer thickness (μm)				1		
Number of layers				150		
Pulse frequency (MHz)				0.67		
Open-circuit voltage (V)				65		
Pulse width (ns)				300		
Current limiting resistance (Ω)				50		

**Table 4:** Machining parameters for Exp. 2.

Parameters	Serial Number			
	a	b	c	d
Tool type	hollow	cylindrical	hollow	cylindrical
Number of layers	800	670	800	670
Rotation speed (rpm)	300		0	
Path type			unidirectional path	
Tool diameter (μm)			800	
Feed speed (μm/s)			500	
Machining length (μm)			1500	
Layer thickness (μm)			1	
Pulse frequency (MHz)			0.04	
Open-circuit voltage (V)			100	
Pulse width (ns)			5000	
Current limiting resistance (Ω)			10	

**Table 5:** Machining parameters for Exp. 3.

Parameters	Serial Number	
	a	b
Tool type	square	cylindrical
Number of layers	150	55
Rotation speed (rpm)		0
Path type		unidirectional path
Tool diameter or edge length (μm)		46
Feed speed (μm/s)		30
Machining length (μm)		500
Layer thickness (μm)		1
Pulse frequency (MHz)		0.67
Open-circuit voltage (V)		65
Pulse width (ns)		300
Current limiting resistance (Ω)		50

**Table 6:** Deviations of the microgroove profile between simulation and Exp. 1.

Deviations	Serial Number					
	a	b	c	d	e	f
Maximum deviation (μm)	4.10	12.17	4.33	3.08	14.93	6.79
Mean deviation (μm)	2.29	5.69	2.85	2.34	7.15	2.14
Mean relative deviation	3.47%	7.90%	3.56%	3.51%	11.09%	2.89%

**Table 7:** Deviations of the microgroove profile between simulation and Exp. 3.

Deviations	Serial Number	
	a	b
Maximum deviation (μm)	16.38	8.22
Mean deviation (μm)	8.02	2.31
Mean relative deviation	10.22%	8.40%

## Data Availability

The data presented in this study are available in this published article.

## Nomenclature

micro-EDM	micro-electrical discharge machining
micro-ECM	micro-electrochemical machining
WEDG	wire electrical discharge grinding
UWM	uniform wear method
LCM	linear compensation method
CLU	method that combined the LCM and UWM
FLC	fixed-length compensation
EB	energy beam
TIF	tool influence function
block-EDG	block electric discharge grinding
*z_T_*	the surface morphologies of the tool electrode
*z_W_*	the surface morphologies of the workpiece
matrix ***D***	the removal depths of *m* points in time and space after *n* pulses
vector ***d****_i_*	the removal depth sequence of point *i*
vector ***d****_j_*	the removal depth sequence of all the points generated by pulse *j*
*x*	*x*-coordinate of the point
*y*	*x*-coordinate of the point
*v*	feed speed (μm/s)
*t*	time (s)
*r*	the distance between the centre point of the bottom surface of the electrode and the point (μm)
*n*	rotation speed (rpm).
Δ*t*	simulation time step (s)
*f*	pulse frequency (Hz)
matrix ***T***	the three-dimensional coordinates of all grid nodes on the tool electrode
matrix ***W***	the three-dimensional coordinates of all grid nodes on the workpiece
matrix ***G***	the distance matrix of point pairs between the tool electrode and the workpiece
*z*_s_	the *z*-coordinate value of the simulated profile
*z*_e_	the *z*-coordinate value of the experimental profile
